# Chaotic Image Encryption Using Hopfield and Hindmarsh–Rose Neurons Implemented on FPGA

**DOI:** 10.3390/s20051326

**Published:** 2020-02-28

**Authors:** Esteban Tlelo-Cuautle, Jonathan Daniel Díaz-Muñoz, Astrid Maritza González-Zapata, Rui Li, Walter Daniel León-Salas, Francisco V. Fernández, Omar Guillén-Fernández, Israel Cruz-Vega

**Affiliations:** 1 Department of Electronics, INAOE, Puebla 72840, Mexico; jdiazm@inaoep.mx (J.D.D.-M.); amgonzalez@inaoep.mx (A.M.G.-Z.); ing.omargufe@gmail.com (O.G.-F.); icruzv@inaoep.mx (I.C.-V.); 2 School of Automation Engineering, UESTC, Chengdu 611731, China; lirui@uestc.edu.cn; 3 School of Engineering Technology, Purdue University, 401 N. Grant St., West Lafayette, IN 47907, USA; wleonsal@purdue.edu; 4 Instituto de Microelectrónica de Sevilla, CSIC and Universidad de Sevilla, 41092 Sevilla, Spain; pacov@imse-cnm.csic.es

**Keywords:** chaos, Hopfield neuron, Hindmarsh–Rose neuron, Lyapunov exponent, image encryption, correlation, FPGA

## Abstract

Chaotic systems implemented by artificial neural networks are good candidates for data encryption. In this manner, this paper introduces the cryptographic application of the Hopfield and the Hindmarsh–Rose neurons. The contribution is focused on finding suitable coefficient values of the neurons to generate robust random binary sequences that can be used in image encryption. This task is performed by evaluating the bifurcation diagrams from which one chooses appropriate coefficient values of the mathematical models that produce high positive Lyapunov exponent and Kaplan–Yorke dimension values, which are computed using TISEAN. The randomness of both the Hopfield and the Hindmarsh–Rose neurons is evaluated from chaotic time series data by performing National Institute of Standard and Technology (NIST) tests. The implementation of both neurons is done using field-programmable gate arrays whose architectures are used to develop an encryption system for RGB images. The success of the encryption system is confirmed by performing correlation, histogram, variance, entropy, and Number of Pixel Change Rate (NPCR) tests.

## 1. Introduction

Image encryption is one of the well-known mechanisms to preserve confidentiality over a reliable unrestricted public channel. However, public channels are vulnerable to attacks and hence efficient encryption algorithms must be developed for secure data transfer. In [1], the authors surveyed ten conventional and five chaos-based encryption techniques to encrypt three test images of different sizes based on various performance metrics, and the important conclusion was that none of the conventional schemes were designed especially for images and hence none of them have any dependence on the initial image. In this manner, the topic of image encryption remains open and several researchers are proposing the use of chaotic systems to mask information that can be transmitted in a secure channel. In this direction, this paper highlights the usefulness of Hopfield and Hindmarsh–Rose neural networks to generate chaotic behavior, and its suitability to design random number generators (RNGs) that are implemented using field-programmable gate arrays (FPGAs).

In [2] J.J. Hopfield introduced the neuron model that nowadays is known as the Hopfield neural network. Ten years later, a modified model of Hopfield neural network was proposed in [3], and applied in information processing. Immediately, the Hopfield neural network was adapted to generate chaotic behavior in [4] where the authors explored bifurcation diagrams. In [5] the simplified Hopfield neuron model was designed to use a sigmoid as activation function, and three neurons were used to generate chaotic behavior. In addition, the authors performed an optimization process updating the weights of the neurons interconnections. The Hopfield neuron was combined with a chaotic map in [6] to be applied in chaotic masking. More recently, the authors in [7] proposed an image encryption algorithm using the Hopfield neural network. In the same direction, the authors in [8] detailed the behavior of Hindmarsh–Rose neuron to generate chaotic behavior. Its bifurcation diagrams were described in [9], and the results were used to select the values of the model to improve chaotic behavior. Hindmarsh–Rose neurons were synchronized in [10], optimizing the scheme of Lyapunov function with two gain coefficients. In this way, the synchronization region is estimated by evaluating the Lyapunov stability. Two Hindmarsh–Rose neurons were synchronized in [11], and the system was used to mask information in continuous time. To show that the neurons generate chaotic behavior, one must compute Lyapunov exponents, and for the Hindmarsh–Rose neuron they were evaluated by the TISEAN package in [12].

The Hopfield neural network has been widely applied in chaotic systems [13,14,15]. This network consists of three neurons, and the authors in [13] proposed a simplified model by removing the synaptic weight connection of the third and second neuron in the original Hopfield network. Numerical simulations were carried out considering values from the bifurcation diagrams, and Lyapunov exponents were evaluated to conclude that the simplified model exhibits rich nonlinear dynamical behaviors including symmetry breaking, chaos, periodic window, antimonotonicity and coexisting self-excited attractors. An FPGA-based modified Hopfield neural network was introduced in [14], to generate multiple attractors, but there are no details of their hardware design from computer arithmetic. The authors in [15] showed the existence of hidden chaotic sets in a simplified Hopfield neural network with three neurons. Similar to the Hopfield neural network, the Hindmarsh–Rose neuron is quite useful, for example: using the Hindmarsh–Rose neuron model, the authors in [16] showed that in the parameter region close to the bifurcation value, where the only attractor of the system is the limit cycle of tonic spiking type, the noise can transform the spiking oscillatory regime to the bursting one. The fractional-order version of the Hindmarsh–Rose neuron was used in [17], for the synchronization of fractional-order chaotic systems. In [18], based on two-dimensional Hindmarsh–Rose neuron and non-ideal threshold memristor, a five-dimensional neuron model of two adjacent neurons coupled by memristive electromagnetic induction, was introduced. In a similar way, the authors in [19] showed the effects of time delay on burst synchronization transitions of a neural network which was locally modeled by Hindmarsh–Rose neurons. On the one hand, the main drawback of those works was the lack of statistical tests according to the National Institute of Standard and Technology (NIST), as done in other chaotic systems given in [20,21,22], to guarantee the randomness of the chaotic sequences. On the other hand, and in addition to NIST tests, the authors in [23] recommend to improve the key space when using chaotic maps, thus enhancing the image encryption schemes. In this work we show the application of neural networks in the design of random number generators (RNGs), whose binary sequences are applied to implement an image encryption scheme [24]. This idea has been previously exploited, for example: the Hopfield neural network was used in [25] to design a RNG, but showed low randomness. In this manner, this paper introduces the selection of the best coefficients of both Hopfield and Hindmarsh–Rose neurons, from the bifurcation diagram, to generate robust chaotic sequences that improve NIST tests and enhance chaotic encryption of images.

Section 2 describes both the Hopfield and the Hindmarsh–Rose neuron models, showing their chaotic behavior. Section 3 shows simulation results of the cases that generate better chaotic time series, applying the 4th-order Runge-Kutta method. Bifurcation diagrams are generated to select appropriate values that improve the generation of chaotic times series that are evaluated using TISEAN, in order to verify the Lyapunov exponents. Section 4 details the FPGA-based implementation of both Hopfield and Hindmarsh–Rose neuron models. Section 5 shows the selection of the series with the highest values of the positive Lyapunov exponent, which are used to generate binary sequences, and whose randomness is evaluated by performing NIST tests. Section 6 shows the application of the generated binary sequences to encrypt an image in a chaotic secure communication system, and the success of the RGB image encryption system is confirmed by performing correlation, histogram, variance, entropy, and Number of Pixel Change Rate (NPCR) tests. Finally, Section 7 summarizes the main results of this work.

## 2. Mathematical Models of Hopfield and Hindmarsh–Rose Neurons

This section describes the mathematical models of both the Hopfield and the Hindmarsh–Rose neural networks. For instance, the complex dynamics of the Hopfield-type neural network with three neurons are analyzed in [26], as well as the observation of the stable points, limit circles, single-scroll chaotic attractors and double-scrolls chaotic attractors. By varying the parameters, the numerical simulations performed in [27] show that the simple Hopfield neural networks can display chaotic attractors and periodic orbits for different parameters, and they associate different values of the Lyapunov exponents and bifurcation plots. The Hindmarsh–Rose neural network is analyzed in [28], and by using the polynomial model previously introduced in [29], the authors perform a detailed bifurcation analysis of the full fast-slow system for bursting patterns.

### 2.1. Hopfield Neuron

The Hopfield neural network can be modeled by Equation (1), where *v* represents the state variables, *c* is a proportional constant, *W* is the weights matrix, and f(v) is associated to the activation function [5].

(1) $$\dot{v}=-cv+Wf\left(v\right).$$

**Figure 1:**
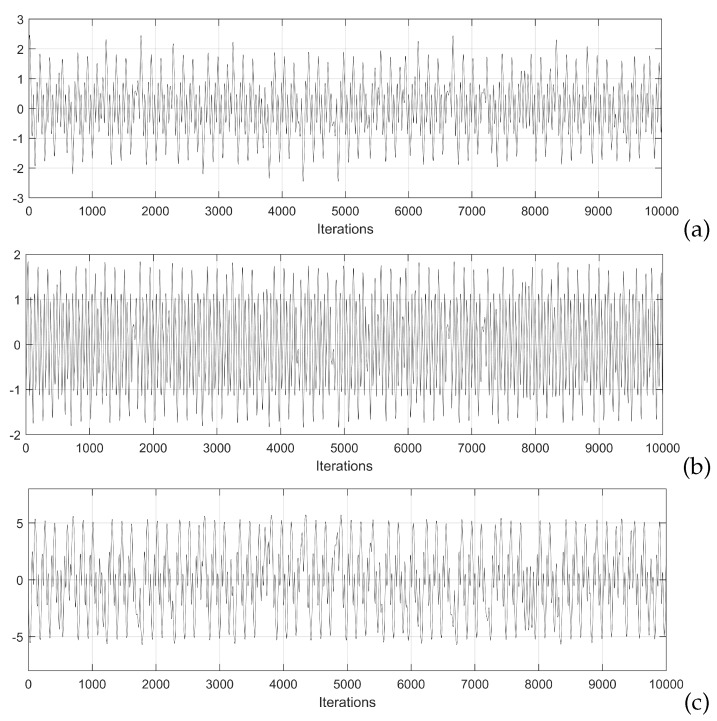
Simulation results of the chaotic time series of the state variables: (**a**) *x*, (**b**) *y*, and (**c**) *z*, of the Hopfield neural network given in [5]. The initial conditions are: $x_{0}$ = 1.951738939809982, $y_{0}$ = −1.207112821944644, and $z_{0}$ = −0.284321234701517.

**Figure 2:**
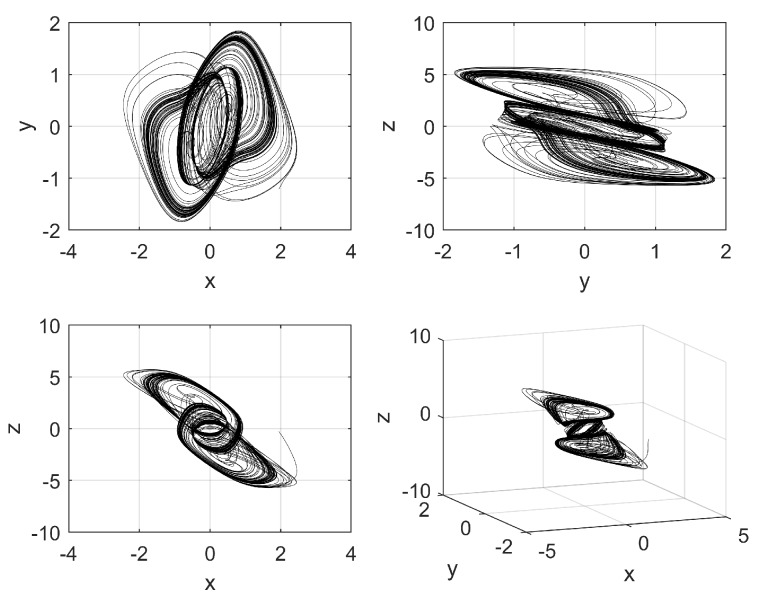
Attractors generated by plotting the state variables shown in Figure 1.

Commonly, *c* is made equal to one, and when a chaotic behavior is desired, the activation function is a hyperbolic tangent function and the weights matrix *W* is modified, whose size depends on the number of neurons. In this work, *W* has size 3 × 3, meaning that the Hopfield neural network has three state variables, associated with each neuron. That way, Equations (2)–(4), describe the model of the three neurons, as shown in [5]. In this case, *v* in (1) is replaced by a three elements vector, including three state variables, namely: *x*, *y*, and *z*; and the control parameter *p* is set to 0.0997. This value is found by exploring values that maximize the positive Lyapunov exponent (LE+). Figure 1 and Figure 2 show the chaotic time series and the attractors obtained by applying the 4th-order Runge–Kutta method.

(2) $$\left[\begin{array}{c} \dot{x}\\ \dot{y}\\ \dot{z} \end{array}\right]=-\left[\begin{array}{c} x\\ y\\ z \end{array}\right]+W\left[\begin{array}{c} f(x)\\ f(y)\\ f(z) \end{array}\right]$$

(3) $$\left[\begin{array}{c} f(x)\\ f(y)\\ f(z) \end{array}\right]=\left[\begin{array}{c} \tanh(x)\\ \tanh(y)\\ \tanh(z) \end{array}\right]$$

(4) $$W=\left[\begin{array}{ccc} 2 & -1.2 & 0\\ 1.9+p & 1.71 & 1.15\\ -4.75 & 0 & 1.1 \end{array}\right].$$

The equilibrium points are obtained by applying the Newton Raphson method $G^{i+1}=G^{i}-{\left[J\left(G_{i}\right)\right]}^{-1}f\left(G_{i}\right)$, where $J(G_{i})$ is the Jacobian, because the neural network has nonlinear terms as tanh(). The Equilibrium points are: $EP_{1}=\left(0,0,0\right),EP_{2}=\left(0.4932,0.3658,-3.2666\right),EP_{3}=\left(-0.4932,-0.3658,3.2666\right)$. The eigenvalues are obtained for each equilibrium point evaluating $\left|\lambda I-J\right|=0$, so that the ones associated to $EP_{1}$ are: $\lambda_{1}$=1.9416 and $\lambda_{2,3}$=−0.0658 ± j 1.8793. The eigenvalues associated to $EP_{2}$ and $EP_{3}$ are: $\lambda_{1}$=−0.9870 and $\lambda_{2,3}$=0.5381 ± j 1.2861.

### 2.2. Hindmarsh–Rose Neuron

The Hindmarsh–Rose neural network can be modeled by three state variables, as given by Equation (5). This model is used to analyze the charge and discharge of a neuron, and in addition, when it provides chaotic behavior, its applications can be extended to cryptography, as shown in this work.

(5) $$\begin{array}{c} \dot{x}=-s\left(-ax^{3}+x^{2}\right)-y-bz\\ \dot{y}=\varphi \left(x^{2}-y\right)\\ \dot{z}=\varepsilon \left(sa_{1}x+b_{1}-kz\right). \end{array}$$

**Figure 3:**
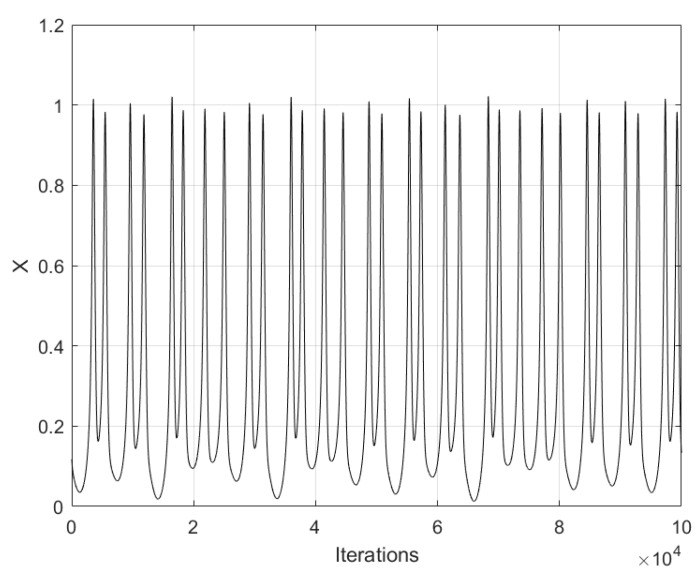
Chaotic time series of state variable *x* of the Hindmarsh–Rose neuron using $x_{0}=0.1169282607$, $y_{0}=0.03563851071$, and $z_{0}=0.01034665217$, as initial conditions.

**Figure 4:**
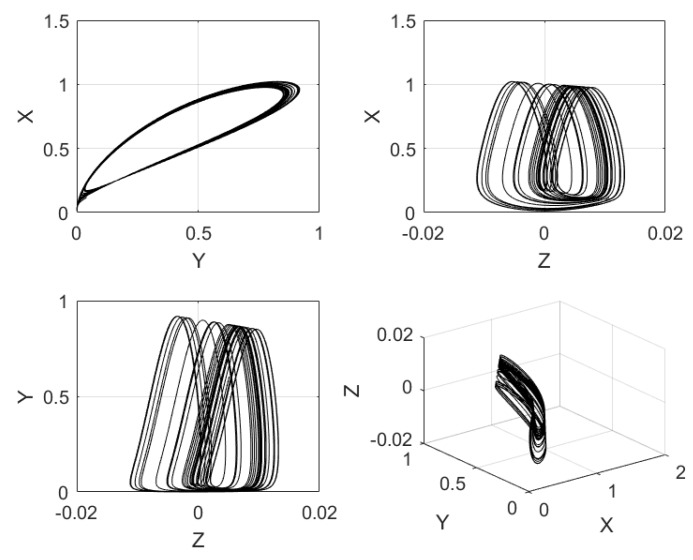
Phase-space portraits of the Hindmarsh–Rose neuron given in Equation (5), using $x_{0}=0.1169282607$, $y_{0}=0.03563851071$, and $z_{0}=0.01034665217$, as initial conditions.

In Equation (5), *x* is associated to the membrane voltage, *y* is the recovery variable associated to the current, and *z* is the slow and adaptable current. The coefficients $a,b,a_{1},b_{1},k,s$ are parameters of the neuron, and φ and ε are associated to the time scale. Their values are set to: φ=1, a=0.5, b=1, $a_{1}=-0.1$, k=0.2, $b_{1}=-0.045$, ε=0.02, and s=−1.605 [11]. Figure 3 shows the time series of the state variable *x* of Hindmarsh–Rose neuron, and Figure 4 shows the phase-space portraits.

The equilibrium points from Equation (5) are: $EP_{1}=\left(0.325817,0.106157,0.0364682\right)$, $EP_{2}=\left(0.214038-j0.902614,-0.7689-j0.38638,-0.053234-j0.7243\right)$, and $EP_{3}=\left(0.214038+j0.902614,-0.7689+j0.38638,-0.053234+j0.7243\right)$. The eigenvalues associated to each equilibrium point are: $\lambda_{1}$ = 0.261784, $\lambda_{2}$ = 0.0204526, $\lambda_{3}$=− 0.495936 for $EP_{1}$; and $\lambda_{1}$ = 2.2075 ± j 1.5659, $\lambda_{2}$=− 0.002488 ±j 0.0001127, and $\lambda_{3}$ = −0.67089 ± j 0.4012, for $EP_{2}$ and $EP_{3}$.

## 3. Bifurcation Diagrams and Selection of the Best Values to Generate Enhanced Chaotic Time Series

**Figure 5:**
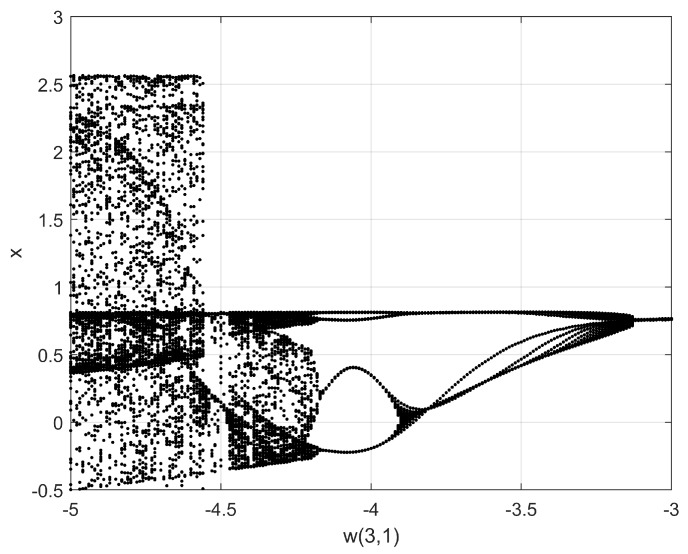
Bifurcation diagram varying W(3,1) in the range given in Table 1.

**Figure 6:**
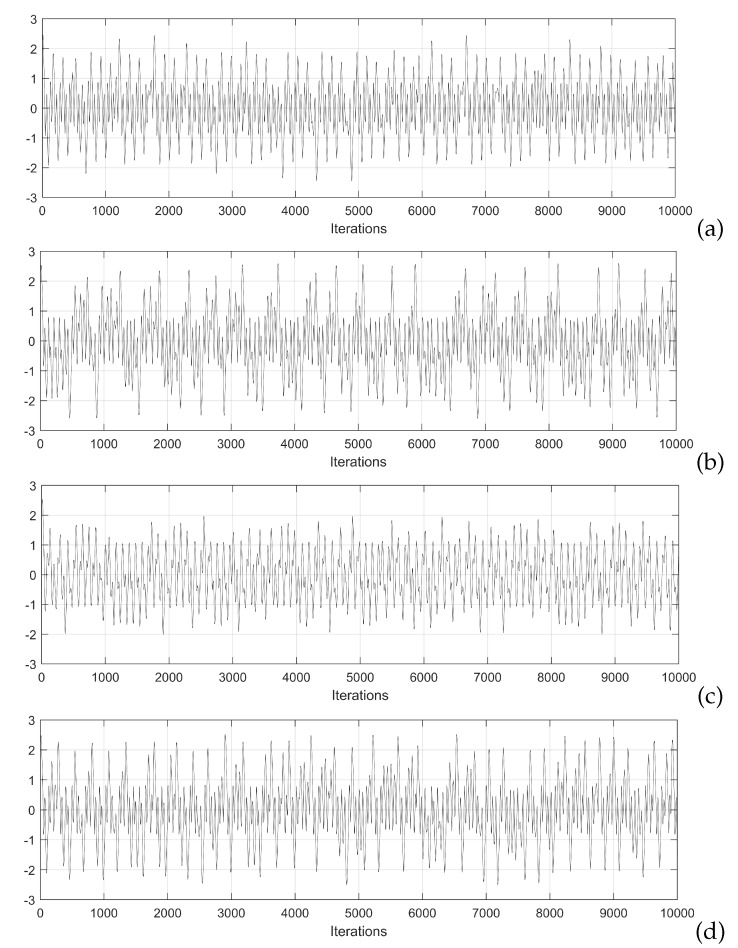
Chaotic time series generated by using the sets of values listed in Table 2: (**a**) original, (**b**) HNNset1, (**c**) HNNset2, and (**d**) HNNset3, plotting the state variable *x*.

Bifurcation diagrams are quite useful to find appropriate values of the mathematical models of the neurons, and in this paper, they are generated to find the best Lyapunov exponents and Kaplan–Yorke dimension, which are considered as appropriate metrics to enhance the generation of chaotic time series. In the case of the Hopfield neuron, the state variable *x* is selected to plot the bifurcation with respect to the control parameter *p*. This process must be performed following the variation of all the coefficients of the mathematical model and for all the state variables in various iterations until some dynamical characteristics of the chaotic system, like the positive Lyapunov exponent (LE+) and Kaplan–Yorke dimension [30], are improved. Varying the weights matrix *W*, one can find better characteristics. For example, in this paper the nine elements in *W* were varied in the ranges and steps listed in Table 1. All these cases generated different bifurcation diagrams from which the feasible values were selected. For example, Figure 5 shows the bifurcation diagram varying W(3,1), where it can be appreciated that the feasible values to generate chaotic behavior must be chosen with values lower than −4.5. In this manner, after exploring the bifurcation diagrams by varying the values in the ranges given in Table 1, three feasible sets of values are given in Table 2, where it can be appreciated their variations with respect to the original values given in [5]. The chaotic time series associated with those sets of values, obtained by applying the 4th-order Runge–Kutta method are shown in Figure 6 for the state variable *x*. Those chaotic time series are used to evaluate Lyapunov exponents and Kaplan–Yorke dimension using TISEAN.

Table 3 lists all Lyapunov exponents values and their associated Kaplan–Yorke dimension for each case from Table 2.

**Figure 7:**
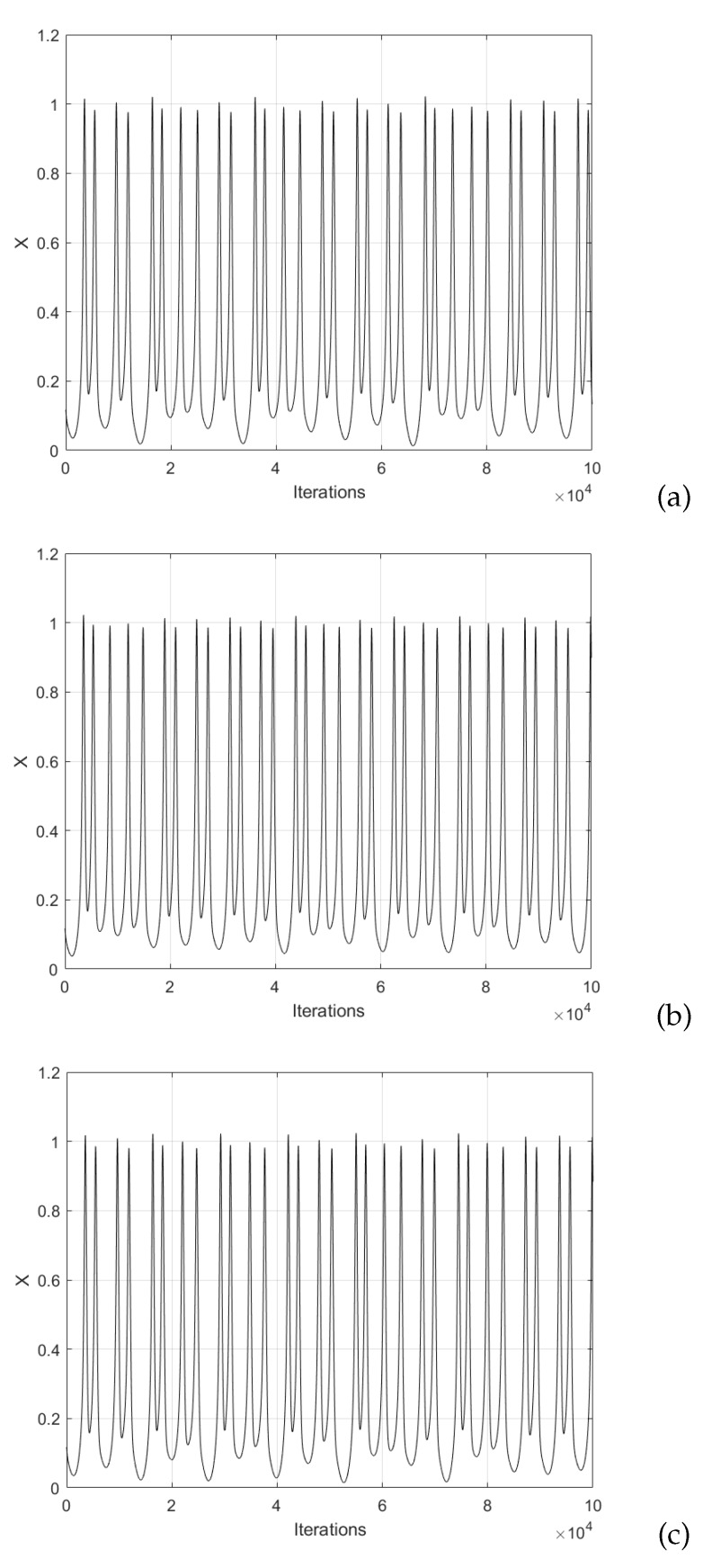
Chaotic time series generated by using the sets of values listed in Table 4: (**a**) original, (**b**) HRNset1, and (**c**) HRNset2, plotting the state variable *x*.

In the case of the Hindmarsh–Rose neural network model given in (5), one can count eight coefficients that can be varied. In this case, a heuristic process was performed with the goal of improving the chaotic behavior. Each coefficient φ, *a*, *b*, $a_{1}$, *k*, $b_{1}$, ε, and *s*, was varied in steps of 0.001 and observing the degradation of chaotic behavior. After performing the variations and observing the bifurcation diagrams, two sets of values were found, which are listed in Table 4. In this manner, Figure 7 shows the chaotic time series associated with these sets of values.

The simulation of the chaotic time series was performed using the initial conditions $x_{0}=0.1169282607$, $y_{0}=0.03563851071$, and $z_{0}=0.01034665217$, and those series were introduced to TISEAN to evaluate Lyapunov exponents and Kaplan–Yorke dimension that are given in Table 5. Since the maximum exponent is positive, then chaotic behavior is guaranteed. In this case, the sets of values HRNset1 is the best because the Kaplan–Yorke dimension is 3, i.e., the ideal value for a three-dimensional dynamical system.

## 4. FPGA-Based Implementation of the Neurons

**Figure 8:**
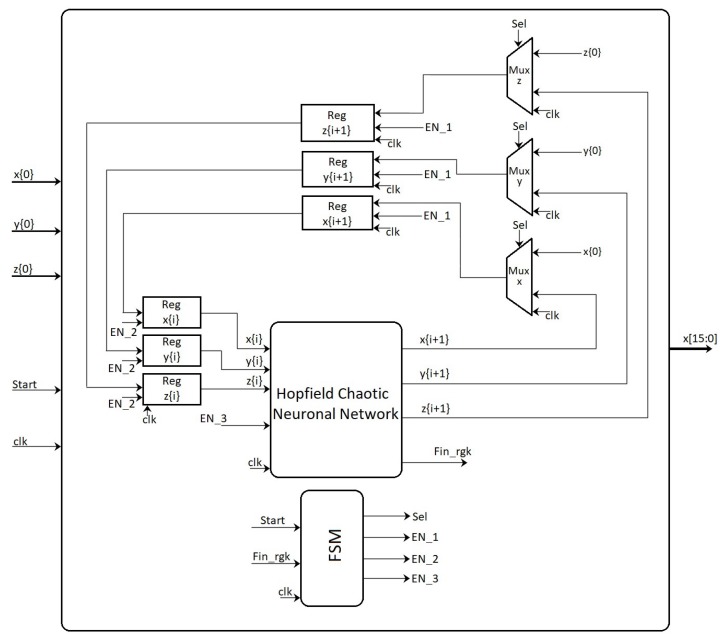
High-level description of the FPGA-based implementation of the Hopfield neural network.

**Figure 9:**
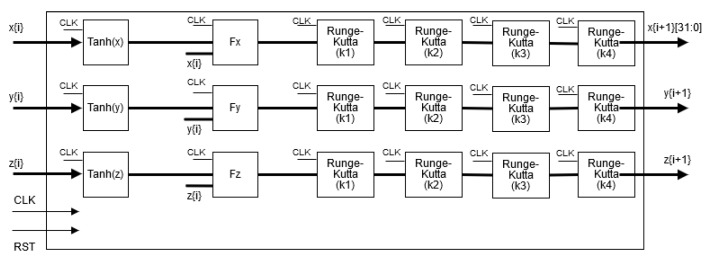
Details of the implementation of the 4th-order Runge–Kutta method solving the Hopfield chaotic neural network, whose block is embedded in Figure 8.

The hardware implementation of both neurons can be performed from the discretized equations using a specific numerical method. For example, in [31] one can find the discretization of a dynamical model by applying Forward Euler and 4th-order Runge–Kutta methods. It can be appreciated that there is a trade-off between exactness and hardware resources. Besides, since the Hopfield neural network is a small dynamical system, the 4th-order Runge–Kutta method is used herein to develop the FPGA-based implementation, as shown in Figure 8, which is based on Equations (2)–(4). The details of the numerical method is sketched in Figure 9, where one can appreciate the block for the hyperbolic tangent function given in Equation (3), which is implemented as already shown in [31]. The general architecture shown in Figure 8 consists of a finite state machine (FSM) that controls the iterations of the numerical method, whose data is saved in the registers (*x*, *y*, *z*). The block labeled Hopfield Chaotic Neuronal Network contains the hardware that evaluates the 4th-order Runge-Kutta method receiving the data at iteration ($x_{i}$, $y_{i}$, $z_{i}$) and providing the data at the next iteration ($x_{i+1}$, $y_{i+1}$, $z_{i+1}$). The Mux blocks introduce the initial conditions ($x_{0}$, $y_{0}$, $z_{0}$) and select the values ($x_{i+1}$, $y_{i+1}$, $z_{i+1}$) for all the remaining iterations. The Reg blocks are parallel-parallel arrays and save the data being processed within the dynamical system. The output of the whole architecture provide a binary string associated to a specific state variable (*x*, *y*, *z*).

The hyperbolic tangent function given in [31] and used in Figure 9, is described by Equations (6) and (7), with L=2, β=1, and θ=0.25. Table 6 shows the hardware resources for the implementation of the four cases given in Table 2 for the Hopfield neuron. The numerical method is the 4th-order Runge–Kutta and the FPGA Cyclone IV EP4CE115F29C7 is used.

(6) $$\mathrm{Tanh}\left(z\right)=G_{s}\left(z\right)=\left\{\begin{array}{cc} \begin{array}{c} 1\\ H_{s}\\ -1 \end{array} & \begin{array}{c} L\leq z\\ -L<z<L\\ z\leq -L \end{array} \end{array}\right.$$

(7) $$H_{s}=\left\{\begin{array}{cc} z(\beta -\theta z) & 0\leq z\leq L\\ z(\beta +\theta z) & -L\leq z\leq 0 \end{array}\right.$$

**Figure 10:**
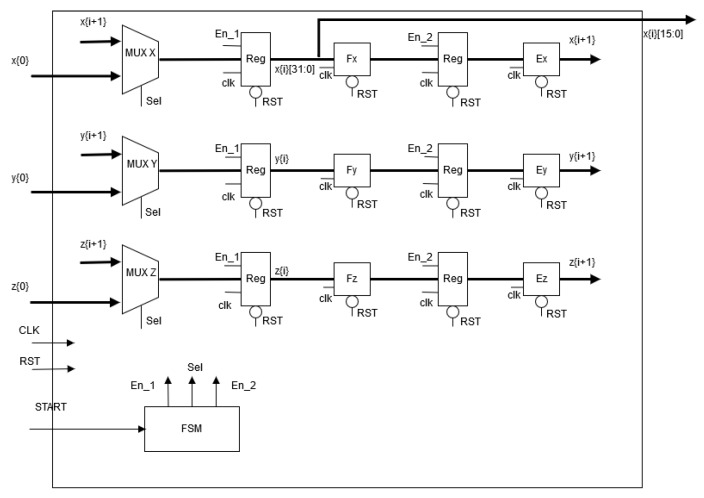
FPGA-based implementation of Hindmarsh–Rose neural network described in Equation (5).

In a similar way, the FPGA-based implementation of Hindmarsh–Rose neural network given in Equation (5), is developed by applying a numerical method. In this case, and applying Forward-Euler method, the hardware description is shown in Figure 10, where it can be appreciated the use of a finite state machine (FSM) to control the iterations associated to the numerical method, the use of multiplexers to process the initial conditions and afterwards the remaining iterations, the use of registers to save the data of the state variables and blocks to evaluate the discretized equations by Forward Euler. The whole iterative process to generate the next value of the state variables requires seven clock (CLK) cycles.

In both FPGA-based implementations for Hopfield and Hindmarsh–Rose neural networks, the computer arithmetic is performed using fixed-point notation of 5.27 for the Hopfield and 3.29 for the Hindmarsh–Rose neural networks. The FPGA resources for the three sets of values of the Hindmarsh–Rose neural network are listed in Table 7.

## 5. Randomness Test: NIST

In this Section, the results of the NIST tests [32,33] for both neural networks are shown. The four cases of Hopfield neurons, and using the state variable *x* with 1000 chaotic time series (binary strings) of 1 million bits each, generated the NIST tests given in Table 8. The results using the original values taken from [5], the three sets of values given in Table 2, and the results using the weight matrix from [14], can be compared. All cases passed NIST tests with proportions around 99%, and the set of values HNNset2 generated a higher *p*-Value average of 0.7065.

The computer arithmetic for the Hindmarsh–Rose neural network is 3.29 but as the largest variation occurs in the least significative bits (LSB), then only the 16 LSB of each 32-bit number were used. The NIST tests were performed using 1000 chaotic time series of 1 million bits each. The results are summarized in Table 9 including the averages of the *p*-Values and proportions.

## 6. Image Encryption Application

**Figure 11:**
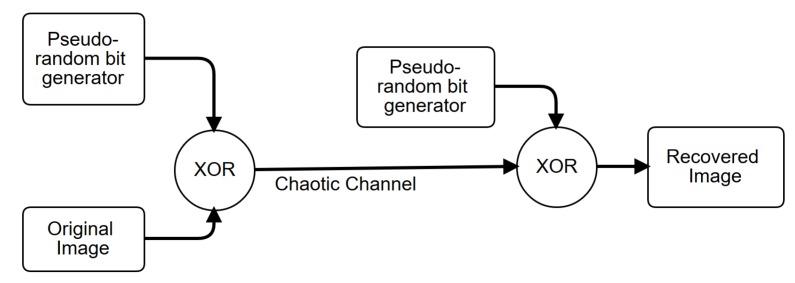
General description of a chaotic secure communication scheme for image encryption based on pseudorandom number generators (PRNGs) implemented by the Hopfield and Hindmarsh–Rose neural networks.

**Figure 12:**
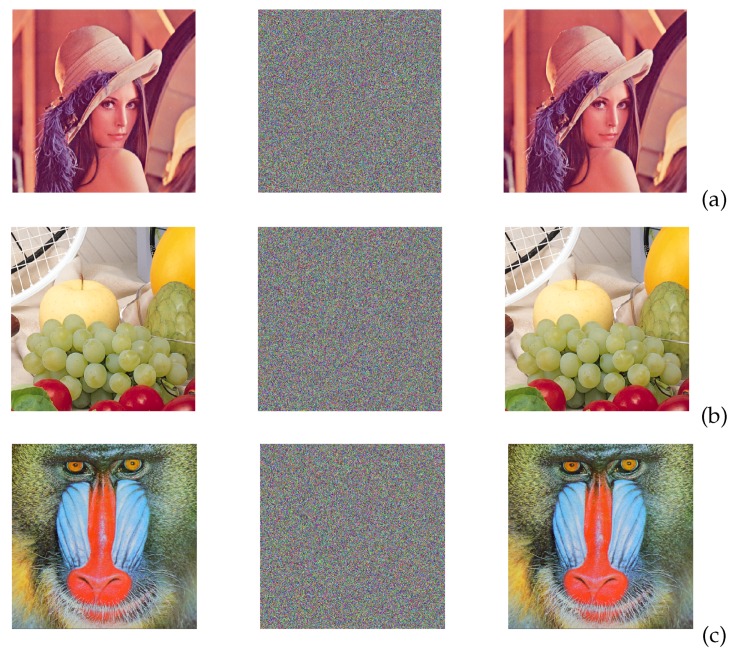
Image encryption using the binary sequences from the FPGA implementation of the Hopfield neuron given in Table 6, and the parameters corresponding to HNNset2. The original image is on the left, the encrypted in the center and the recovered on the right column, for: (**a**) Lena, (**b**) Fruits, and (**c**) Baboon images.

The binary sequences tested in the previous section can be taken as pseudorandom number generators (PRNGs), and can be used to design a chaotic secure communication system to encrypt images, as shown in Figure 11. Those PRNGs can be implemented by either the Hopfield or Hindmarsh–Rose neural networks because both provide high randomness. Both neurons can also be implemented using memristors, as shown in [34], which constitutes another research direction of hardware security. For instance, using the four binary sequences from the FPGA-based implementation of the Hopfield neural network, we show the encryption of three images (Lena, Fruits and Baboon) in Figure 12. The three RGB images have a resolution of 512 × 512 pixels.

The correlation analysis is performed using [35]: Equation (8)–(10), and it provides the values given in Table 10. The first row x,y,z, means that the chaotic time series of state *x* is used to encrypt R (red), *y* to G (green) and *z* to B (blue). The second row means that all R, G and B are encrypted using the data from *x*, and so on.

(8) $$r_{xy}=\frac{E\left\{\left(x-E(x)\right)\left(y-E(y)\right)\right\}}{\left(\sqrt{D(x)}\right)\left(\sqrt{D(y)}\right)}=\frac{cov(x,y)}{\left(\sqrt{D(x)}\right)\left(\sqrt{D(y)}\right)}$$

(9) $$E\left(x\right)=\frac{1}{S}\sum_{i=1}^{S}x_{i}$$

(10) $$E\left(x\right)=\frac{1}{S}\sum_{i=1}^{S}{\left(x_{i}-E\left(x\right)\right)}^{2}.$$

**Figure 13:**
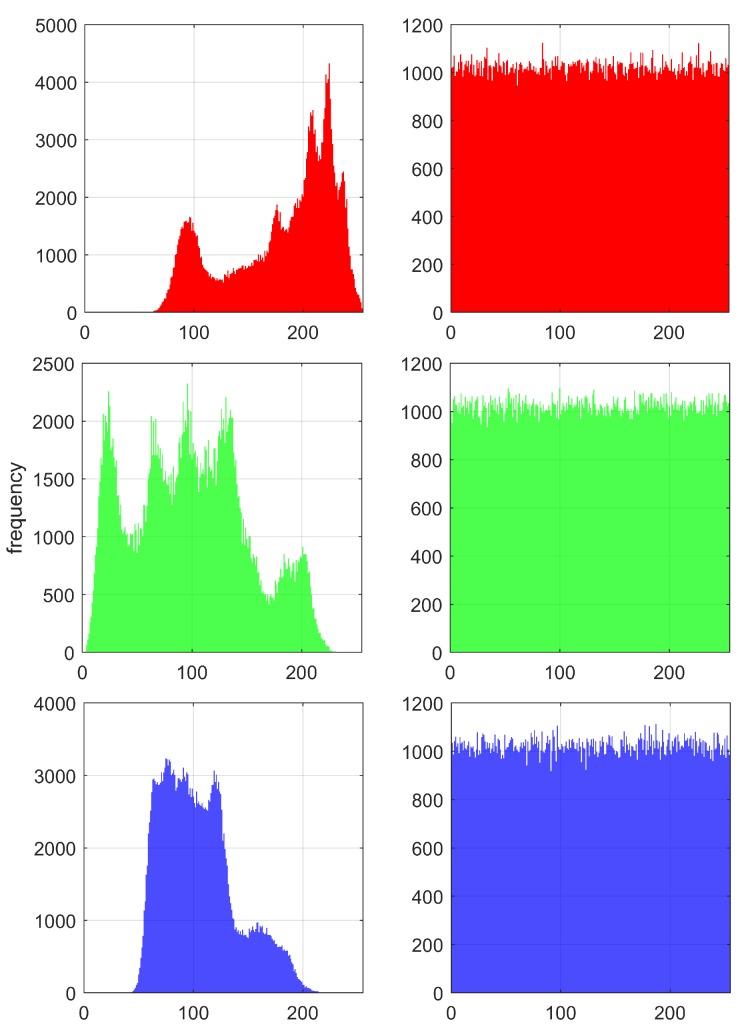
Histograms of the Lena image encrypted using HNNset2 from Table 2. Original images on the left and the R, G and B encryption on the right.

Figure 13 shows the histograms of Lena before and after encryption using HNNset2 from Table 2. To describe the distribution characteristics of the histograms quantitatively, the variance of the histograms for the three images (Lena, Fruits and Baboon) is calculated according to [36], and the results are shown in Table 11.

The entropy is evaluated by Equation (11), where $P(s_{i})$ represents the probability of the datum $s_{i}$. Using 8 bits (N=8), Table 12 shows the entropies for the three images (Lena, Fruits and Baboon).

(11) $$H\left(s\right)=\sum_{i=0}^{2^{N}-1}P\left(s_{i}\right)log_{2}\frac{1}{P(s_{i})}bits.$$

To verify the encryption capability against differential attacks, the NPCR test is evaluated by Equations (12) and (13) [37], where $C^{1}\left(i,j\right)$ and $C^{2}\left(i,j\right)$ are two cipher images that are encrypted from two plain images with only one-bit difference. In this case, using HNNset2 for the Lena, Fruits and Baboon images, the NPCR values are: 99.2672 when using state variable *x*, 99.2886 when using the state variable *y*, and 99.3420 when using *z*. All NPCR results pass the criterium given in [38].

(12) $$NPCR=\sum_{ij}\frac{D(i,j)}{T}\times 100\%$$

(13) $$D\left(i,j\right)=\left\{\begin{array}{cc} \begin{array}{c} 0\\ 1 \end{array} & \begin{array}{c} C^{1}\left(i,j\right)=C^{2}\left(i,j\right)\\ C^{1}\left(i,j\right)\neq C^{2}\left(i,j\right) \end{array} \end{array}\right..$$

**Figure 14:**
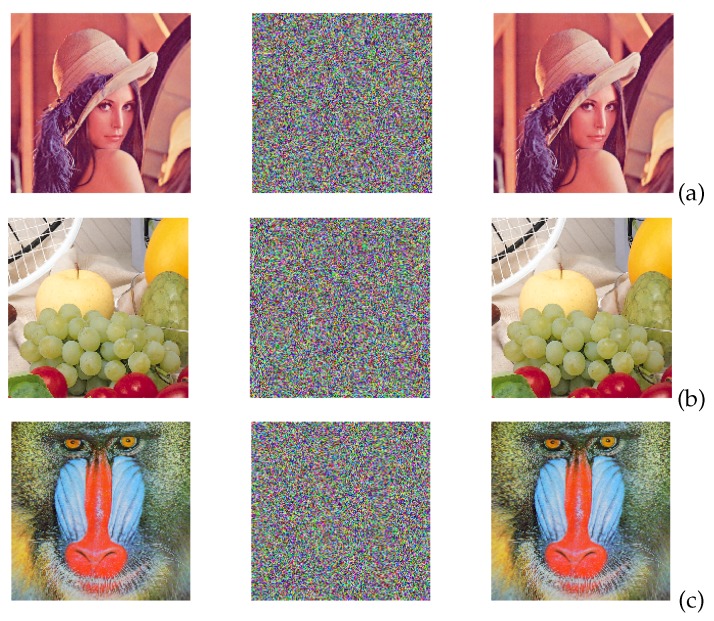
Image encryption of (**a**) Lena, (**b**) Fruits and (**c**) Baboon, using the binary sequences from the FPGA implementation of Hindmarsh–Rose neuron with HRNset1 from Table 7. The original image is on the left, the encrypted in the center and the recovered on the right column.

The binary sequences from the FPGA implementation of the Hindmarsh–Rose neural network shown in Figure 7, were used as PRNG to encrypt the Lena, Fruits and Baboon images, which results are shown in Figure 14.

The correlation analysis between the images and the sets of values from Table 7, provides the values given in Table 13. The first row x,y,z, means that the chaotic time series of state *x* is used to encrypt R (red), *y* to G (green) and *z* to B (blue). The second row means that all R, G and B are encrypted using the data from *x*, and so on.

**Figure 15:**
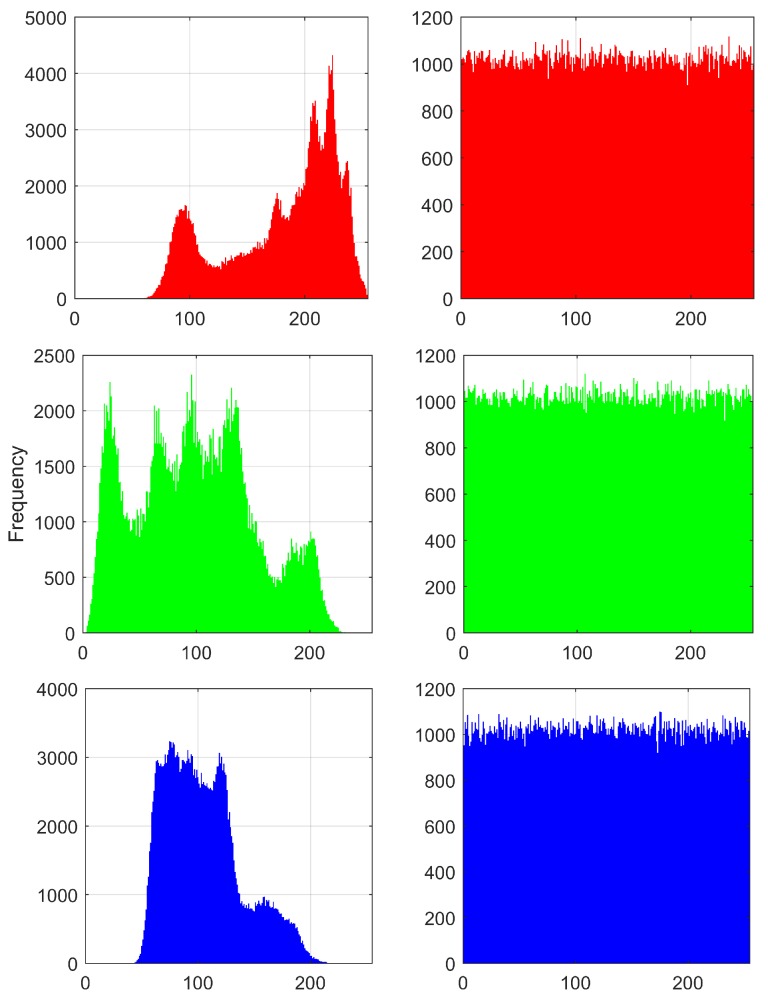
Histograms of the Lena image encrypted using the set of values HRNset1. Original images on the left and the R, G and B encryption on the right.

Figure 15 shows the histogram of the Lena image before and after encryption using HRNset1 from Table 4. The variance of the histograms for the three images (Lena, Fruits and Baboon) using HRNset1, is calculated according to [36], and the results are shown in Table 14.

The entropy is evaluated by (11) using HRNset1, and using 8 bits (N=8), Table 15 shows the entropies for the three images (Lena, Fruits and Baboon).

The key space is equal to 160 bits because each datum is encoded by 32 bits, the initial condition for each state variable counts, the step-size of the numerical method can change and is also encoded using 32 bits. The NPCR tests results using HRNset1 for the color images, were: 99.60365 when using the state variable *x*, 99.49608 when using the state variable *y*, and 98.49586 when using the state variable *z*.

In both FPGA-based implementations for the Hopfield and Hindmarsh–Rose neural networks, the image is transmitted from a personal computer running MatLab to the FPGA using the serial port RS-232, as described in [31].

## 7. Conclusions

The use of two well-known neural networks, the Hopfield and Hindmarsh–Rose ones, for image encryption applications has been described. With the help of bifurcation diagrams, new feasible sets of values were proposed in order to generate binary strings with more randomness than the ones previously published in the literature. We proposed three sets of values for the Hopfield neuron and two sets of values for the Hindmarsh–Rose neuron. The chaotic time series were analyzed by TISEAN to compute Lyapunov exponents and Kaplan–Yorke dimension. The proposed sets of values were much better than the already published ones.

By applying numerical methods, we showed the descriptions of the hardware design of both neurons and the FPGA resources were listed for the Hopfield and Hindmarsh–Rose neurons, respectively. The binary strings that were generated by the FPGA-based implementations of both neurons were taken as PRNGs to perform the encryption of the RGB Lena, Fruits and Baboon images. The success of the encryption system has been confirmed by the results obtained from correlation, histogram, variance, entropy, and NPCR tests. This demonstrates that both neurons are very useful for chaotic image encryption.

## Figures and Tables

**Table 1:** Variation conditions of the elements in *W*.

Matrix Element	Variation Range	Step
W(1,1)	[−3, 3]	0.01
W(1,2)	[−2, 2]	0.01
W(1,3)	[−0.5, 0.5]	0.01
W(2,1)	[−2.9, 2.9]	0.01
W(2,2)	[−2.6, 2.6]	0.01
W(2,3)	[−1.7, 1.7]	0.01
W(3,1)	[−5, 5]	0.01
W(3,2)	[−0.5, 0.5]	0.01
W(3,3)	[−1.9, 1.9]	0.01

**Table 2:** Proposed sets of values to generate enhanced chaotic behavior using Hopfield neuron.

Matrix Element	Sets of Values			
	**Original [5]**	**HNNset1**	**HNNset2**	**HNNset3**
W(1,1)	2	2	2	1.98
W(1,2)	−1.2	−1.26	−1.26	−1.18
W(1,3)	0	0	0	0.01
W(2,1)	1.9997	1.93	1.93	1.94
W(2,2)	1.71	1.71	1.71	1.71
W(2,3)	1.15	1.2	1.2	1.2
W(3,1)	−4.75	−4.76	−4.76	−4.74
W(3,2)	0	0.06	0.06	0.03
W(3,3)	1.1	1.1	−0.11	1.1

**Table 3:** Lyapunov exponents and Kaplan–Yorke dimension $D_{KY}$ associated to each set of values from Table 2.

Sets of Values	Lyapunov Exponents			$D_{\mathrm{KY}}$
Original [5]	$9.443281\times 10^{-3}$	$-2.407246\times 10^{-3}$	$-6.072266\times 10^{-2}$	2.115872
HNNset1	$1.769144\times 10^{-2}$	$-2.681429\times 10^{-3}$	$-8.082816\times 10^{-2}$	2.185703
HNNset2	$1.294477\times 10^{-2}$	$-1.194553\times 10^{-5}$	$-7.149536\times 10^{-2}$	2.164349
HNNset3	$1.488237\times 10^{-2}$	$-9.456641e\times 10^{-4}$	$-7.905735\times 10^{-2}$	2.176286

**Table 4:** Proposed sets of values to generate enhanced chaotic behavior using the Hindmarsh–Rose neuron.

Coefficient	Sets of Values		
	**Original [11]**	**HRNset1**	**HRNset2**
*a*	0.5	0.5	0.5
*b*	1	0.99	1.005
φ	1	1	1
$a_{1}$	−0.1	−0.095	−0.1
*k*	0.2	0.15	0.198
$b_{1}$	−0.045	−0.045	−0.045
ε	0.02	0.02	0.02
*s*	−1.605	−1.610	−1.607

**Table 5:** Lyapunov exponents and Kaplan–Yorke dimension $D_{KY}$ associated to each set of values from Table 4.

Sets of Values	Lyapunov Exponents			$D_{\mathrm{KY}}$
Original [11]	$6.212\times 10^{-3}$	$-6.2947\times 10^{-5}$	$-6.3243\times 10^{-3}$	2.9723
HRNset1	$7.3873\times 10^{-3}$	$6.5024\times 10^{-4}$	$-7.7942\times 10^{-3}$	3.0000
HRNset2	$3.5572\times 10^{-3}$	$-1.7318\times 10^{-4}$	$-5.2275\times 10^{-3}$	2.6473

**Table 6:** FPGA resources for the implementation of the Hopfield neuron from the sets of values given in Table 2.

Resources	FPGA Cyclone IV EP4CE115F29C7 and 4th-order Runge-Kutta				Available
	Original [5]	HNNset1	HNNset2	HNNset3	
Logic Elements	5051	5194	5174	5333	114,480
Registers	3979	4037	4033	4091	114,480
9 × 9 bits Multipliers	128	136	136	144	532
Maximum Frequency (MHz)	55.55	56.14	57.04	55.17	50
Clock Cycles by Iteration	40	40	40	40	-
Latency by Iteration (ns)	800	800	800	800	-

**Table 7:** FPGA resources for the implementation of the Hindmarsh–Rose neuron from the sets of values given in Table 4.

Resources	FPGA Cyclone IV EP4CE115F29C7 and Forward Euler			Avaible
	Original [11]	HRNset1	HRNset2	
Logic Elements	2161	2172	2171	114,480
Registers	1173	1173	1173	114,480
9×9 bits multipliers	104	104	104	532
Maximun Frequency (MHz)	91.73	94.23	92.46	50
Clock cycles by iteration	9	9	9	−−
Latency by iteration (ns)	180	180	180	−−

**Table 8:** National Institute of Standard and Technology (NIST) tests for the binary sequences generated by the Hopfield neural network for the state variable *x*, and for the sets of values given in Table 2.

Test	Original [5]		HNNset1		HNNset2		HNNset3		[14]	
	*p*-Value	Proportion	*p*-Value	Proportion	*p*-Value	Proportion	*p*-Value	Proportion	*p*-Value	Proportion
Frequency (Monobit)	0.6911	991/1000	0.1876	992/1000	0.7339	988/1000	0.4391	989/1000	0.3115	992/1000
Frequency Test within a Block	0.1969	991/1000	0.4318	992/1000	0.5544	992/1000	0.3686	992/1000	0.7617	993/1000
Runs	0.5606	991/1000	0.0533	987/1000	0.9830	988/1000	0.1031	993/1000	0.2596	991/1000
Longest-Run-of Ones in a Block	0.0046	994/1000	0.4466	988/1000	0.7887	986/1000	0.3457	988/1000	0.7014	990/1000
Binary Matrix Rank	0.4769	993/1000	0.5544	991/1000	0.7096	984/1000	0.2854	991/1000	0.6683	993/1000
Discrete Fourier Transform	0.7238	989/1000	0.5585	990/1000	0.2911	987/1000	0.4136	985/1000	0.0915	987/1000
Non-overlapping Template Matching	0.7868	989/1000	0.9997	988/1000	0.9958	988/1000	0.9858	992/1000	0.9958	993/1000
Overlapping Template Matching	0.1274	983/1000	0.8183	989/1000	0.2356	987/1000	0.4336	989/1000	0.4428	997/1000
Maurer’s “Universal Statistical”	0.0183	987/1000	0.1478	989/1000	0.7519	984/1000	0.7558	986/1000	0.0323	992/1000
Linear Complexity	0.9191	990/1000	0.9825	995/1000	0.5362	991/1000	0.5852	988/1000	0.8183	988/1000
Serial	0.8378	993/1000	0.6434	990/1000	0.7676	989/1000	0.4318	989/1000	0.4172	997/1000
Approximate Entropy	0.8645	990/1000	0.0236	989/1000	0.3703	990/1000	0.7399	993/1000	0.4559	995/1000
Cumulative Sums	0.4788	993/1000	0.3686	991/1000	0.9432	992/1000	0.3703	991/1000	0.6309	991/1000
Random Excursions	0.7151	610/615	0.7514	607/615	0.9867	616/622	0.8566	613/617	0.8670	641/647
Random Excursions Variant	0.5491	611/615	0.9103	610/615	0.9501	617/622	0.8400	611/617	0.8019	640/647
Average	0.5301	991/1000	0.5252	990/1000	0.7065	987/1000	0.5303	990/1000	0.5504	992/1000

**Table 9:** NIST tests for the binary sequences generated by the Hindmarsh–Rose neural network for the state variable *x*, and for the sets of values given in Table 4.

Test	Original [11]		HRNset1		HRNset2	
	*p*-Value	Proportion	*p*-Value	Proportion	*p*-Value	Proportion
Frequency (Monobit)	0.33297	995/1000	0.032923	996/1000	0.04037	989/1000
Frequency Test within a Block	0.01188	976/1000	0.39246	983/1000	0.00003	985/1000
Cumulative Sums	0.20119	991/1000	0.87708	992/1000	0.72178	991/1000
Runs	0.33141	985/1000	0.85964	988/1000	0.21557	989/1000
Longest-Run-of-Ones in a Block	0.56055	993/1000	0.78493	993/1000	0.04090	988/1000
Binary Matrix Rank	0	950/1000	0	964/1000	0	970/1000
Discrete Fourier Transform	0.20663	984/1000	0.50817	992/1000	0.09543	984/1000
Non-Overlapping Template Matching	0.98579	994/1000	0.99323	992/1000	0.99715	991/1000
Overlapping Template Matching	0.32521	985/1000	0.27846	989/1000	0.02980	993/1000
Maurer’s “Universal Statistical”	0.01068	979/1000	0.82372	985/1000	0.74790	979/1000
Approximate Entropy	0.71160	996/1000	0.48077	985/1000	0.47691	989/1000
Random Excursions	0.82551	595/604	0.70188	636/648	0.69967	580/587
Random Excursions Variant	0.99464	599/604	0.95355	642/648	0.87820	580/587
Serial	0.58727	991/1000	0.44656	994/1000	0.80187	984/1000
Linear Complexity	0.91532	990/1000	0.74591	988/1000	0.25312	989/1000
Average	0.46671	985/1000	0.59195	987/1000	0.39991	986/1000

**Table 10:** Correlations between the chaotic channel and the RGB Lena, Fruits and Baboon images using the sequences generated by the Hopfield neuron for the state variables *x*, *y*, and *z*.

Image	Encryption	Sets of Values			
		Original [5]	HNNset1	HNNset2	HNNset3
**Lena**	x,y,z	0.00108	0.00140	−0.00054	0.00094
	*x*	−0.00221	−0.00257	0.00058	−0.00278
	*y*	−0.00139	0.00062	0.00195	0.00483
	*z*	0.00041	0.00150	−0.00119	0.00245
**Fruits**	x,y,z	−0.00036	−0.00073	−0.00055	0.00085
	*x*	0.00215	−0.00293	−0.00050	−0.00026
	*y*	−0.00216	0.00003	0.00076	0.00359
	*z*	0.00175	−0.00026	−0.00110	0.00288
**Baboon**	x,y,z	0.00008	−0.00015	−0.00062	0.00079
	*x*	−0.00052	−0.00255	−0.00087	−0.00269
	*y*	−0.00125	0.00030	0.00167	0.00157
	*z*	0.00176	0.00049	−0.00079	0.00203

**Table 11:** Variance of the histograms for the Lena, Fruits and Baboon images using the sequences generated by HNNset2.

Encryption	Color	Variance					
		Lena		Fruits		Baboon	
		Original Image	HNNset2	Original Image	HNNset2	Original Image	HNNset2
x,y,z	R	1,017,334.70	824.77	2,559,535.12	1005.51	331,358.91	1014.40
	G	455,718.80	965.59	1,131,488.32	930.56	571,232.16	934.05
	B	1,377,355.87	1071.79	338280.76	891.06	319,770.47	1178.41
*x*	R	1,017,334.70	824.77	2,559,535.12	1005.51	331,358.91	1014.40
	G	455,718.80	1033.72	1,131,488.32	1096.43	571,232.16	1017.02
	B	1,377,355.87	798.66	338,280.76	1233.43	319,770.47	1064.48
*y*	R	1,017,334.70	1188.50	2,559,535.12	963.14	331,358.91	1064.51
	G	455,718.80	965.59	1,131,488.32	930.56	571,232.16	934.05
	B	1,377,355.87	1107.14	338,280.76	1026.64	319,770.47	1058.48
*z*	R	1,017,334.70	1079.58	2,559,535.12	1136.72	331,358.91	1261.09
	G	455,718.80	1055.34	1,131,488.32	1033.53	571,232.16	1133.23
	B	1,377,355.87	1071.79	338,280.76	891.06	319,770.47	1178.41

**Table 12:** Entropies of the original Lena, Fruits and Baboon images and the encrypted ones using the sets of values HNNset2 from Table 2.

Encryption	Color	Entropy					
		Lena		Fruits		Baboon	
		Original Image	HNNset2	Original Image	HNNset2	Original Image	HNNset2
x,y,z	R	7.2531	7.9994	7.0556	7.9994	7.7067	7.9993
	G	7.5940	7.9993	7.3527	7.9994	7.4744	7.9994
	B	6.9684	7.9993	7.7134	7.9993	7.7522	7.9992
*x*	R	7.2531	7.9994	7.0556	7.9993	7.7067	7.9993
	G	7.5940	7.9993	7.3527	7.9992	7.4744	7.9993
	B	6.9684	7.9995	7.7134	7.9992	7.7522	7.9993
*y*	R	7.2531	7.9992	7.0556	7.9993	7.7067	7.9993
	G	7.5940	7.9993	7.3527	7.9994	7.4744	7.9994
	B	6.9684	7.9992	7.7134	7.9993	7.7522	7.9993
*z*	R	7.2531	7.9993	7.0556	7.9992	7.7067	7.9991
	G	7.5940	7.9993	7.3527	7.9993	7.4744	7.9992
	B	6.9684	7.9993	7.7134	7.9994	7.7522	7.9992

**Table 13:** Correlations between the chaotic channel and the RGB Lena, Fruits and Baboon images using the sequences generated by the Hindmarsh–Rose neuron for the state variables *x*, *y*, and *z*.

Image	Encryption	Sets of Values		
		Original [11]	HRNset1	HRNset2
**Lena**	x,y,z	0.00016	0.00002	0.00147
	*x*	0.00083	0.00075	0.00110
	*y*	0.00093	0.00010	0.00205
	*z*	0.00008	0.00314	0.00024
**Fruits**	x,y,z	0.00007	0.00066	0.00011
	*x*	0.00291	0.00245	0.00001
	*y*	0.00036	0.00207	0.00328
	*z*	0.00162	0.00307	0.00089
**Baboon**	x,y,z	0.00078	0.00102	0.00160
	*x*	0.00084	0.00069	0.00206
	*y*	0.00120	0.00002	0.00119
	*z*	0.00016	0.00414	0.00111

**Table 14:** Variance of the histograms for the Lena, Fruits and Baboon images using the sequences generated by HRNset1.

Encryption	Color	Variance					
		Lena		Fruits		Baboon	
		Original Image	HRNset1	Original Image	HRNset1	Original Image	HRNset1
x,y,z	R	1017334.60	969.95	2,559,535.12	954.50	331,358.90	996.01
	G	455,718.80	1002.58	1,131,488.32	1020.69	571,232.15	1051.16
	B	1,377,355.80	995.09	338,280.76	904.91	319,770.47	1165.13
*x*	R	1,017,334.60	969.95	2,559,535.12	954.50	331,358.90	996.01
	G	455,718.80	1189.90	1,131,488.32	997.30	571,232.15	1143.03
	B	1,377,355.80	1126.64	338,280.76	914.46	319,770.47	1071.51
*y*	R	1,017,334.60	1005.61	2,559,535.12	943.03	331,358.90	1109.45
	G	455,718.80	1002.58	1,131,488.32	1020.69	571,232.15	1051.16
	B	1,377,355.80	1025.56	338,280.76	1025.20	319,770.47	940.07
*z*	R	1,017,334.60	1053.77	2,559,535.12	1044.60	331,358.90	1175.52
	G	455,718.80	1309.57	1,131,488.32	1154.54	571,232.15	1008.48
	B	1,377,355.80	995.09	338,280.76	904.91	319,770.47	1165.13

**Table 15:** Entropies of the original Lena, Fruits and Baboon images and the encrypted ones using the sets of values HRNset1 from Table 4.

Encryption	Color	Entropy					
		Lena		Fruits		Baboon	
		Original Image	HRNset1	Original Image	HRNset1	Original Image	HRNset1
x,y,z	R	7.2531	7.9993	7.0556	7.9993	7.7067	7.9993
	G	7.5940	7.9993	7.3527	7.9993	7.4744	7.9993
	B	6.9684	7.9993	7.7134	7.9994	7.7522	7.9992
*x*	R	7.2531	7.9993	7.0556	7.9993	7.7067	7.9993
	G	7.5940	7.9992	7.3527	7.9993	7.4744	7.9992
	B	6.9684	7.9992	7.7134	7.9994	7.7522	7.9993
*y*	R	7.2531	7.9993	70556	7.9994	7.7067	7.9992
	G	7.594	7.9993	7.3527	7.9993	7.4744	7.9993
	B	6.9684	7.9993	7.7134	7.9993	7.7522	7.9994
*z*	R	7.2531	7.9993	7.0556	7.9993	7.7067	7.9992
	G	7.5940	7.9991	7.3527	7.9992	7.4744	7.9993
	B	6.9684	7.9993	7.7134	7.9994	7.7522	7.9992

## References

[1] Kumari M., Gupta S., Sardana P. (2017). A Survey of Image Encryption Algorithms. 3D Res..

[2] Hopfield J.J. (1982). Neural networks and physical systems with emergent collective computational abilities. Proc. Natl. Acad. Sci. USA.

[3] Nozawa H. (1992). A neural network model as a globally coupled map and applications based on chaos. Chaos Interdiscip. J. Nonlinear Sci..

[4] Chen L., Aihara K. (1995). Chaotic simulated annealing by a neural network model with transient chaos. Neural Netw..

[5] Yang X.S., Yuan Q. (2005). Chaos and transient chaos in simple Hopfield neural networks. Neurocomputing.

[6] Yu W., Cao J. (2006). Cryptography based on delayed chaotic neural networks. Phys. Lett. A.

[7] Wang X.Y., Li Z.M. (2019). A color image encryption algorithm based on Hopfield chaotic neural network. Secur. Commun. Netw..

[8] Osipov V.V., Ponizovskaya E.V. (1998). The nature of bursting noises, stochastic resonance and deterministic chaos in excitable neurons. Phys. Lett. A.

[9] Storace M., Linaro D., de Lange E. (2008). The Hindmarsh–Rose neuron model: Bifurcation analysis and piecewise-linear approximations. Chaos Interdiscip. J. Nonlinear Sci..

[10] Wang C., He Y., Ma J., Huang L. (2014). Parameters estimation, mixed synchronization, and antisynchronization in chaotic systems. Complexity.

[11] Wang T., Wang D., Wu K. (2018). Chaotic Adaptive Synchronization Control and Application in Chaotic Secure Communication for Industrial Internet of Things. IEEE Access.

[12] Hegger R., Kantz H., Schreiber T. (1999). Practical implementation of nonlinear time series methods: The TISEAN package. Chaos Interdiscip. J. Nonlinear Sci..

[13] Njitacke Z.T., Kengne J. (2019). Nonlinear Dynamics of Three-Neurons-Based Hopfield Neural Networks (HNNs): Remerging Feigenbaum Trees, Coexisting Bifurcations and Multiple Attractors. J. Circ. Syst. Comput..

[14] Rajagopal K., Munoz-Pacheco J.M., Pham V.T., Hoang D.V., Alsaadi F.E., Alsaadi F.E. (2018). A Hopfield neural network with multiple attractors and its FPGA design. Eur. Phys. J. Spec. Top..

[15] Danca M.F., Kuznetsov N. (2017). Hidden chaotic sets in a Hopfield neural system. Chaos Solitons Fractals.

[16] Bashkirtseva I., Ryashko L., Slepukhina E. (2019). Noise-induced spiking-bursting transition in the neuron model with the blue sky catastrophe. Phys. Rev. E.

[17] Vafaei V., Kheiri H., Akbarfam A.J. (2019). Synchronization of fractional-order chaotic systems with disturbances via novel fractional-integer integral sliding mode control and application to neuron models. Math. Methods Appl. Sci..

[18] Bao H., Liu W., Hu A. (2019). Coexisting multiple firing patterns in two adjacent neurons coupled by memristive electromagnetic induction. Nonlinear Dyn..

[19] Sun X., Xue T. (2018). Effects of Time Delay on Burst Synchronization Transition of Neuronal Networks. Int. J. Bifurc. Chaos.

[20] Angel Murillo-Escobar M., Omar Meranza-Castillon M., Martha Lopez-Gutierrez R., Cruz-Hernandez C. (2019). Suggested Integral Analysis for Chaos-Based Image Cryptosystems. Entropy.

[21] Nesa N., Ghosh T., Banerjee I. (2019). Design of a chaos-based encryption scheme for sensor data using a novel logarithmic chaotic map. J. Inf. Secur. Appl..

[22] Ding L., Liu C., Zhang Y., Ding Q. (2019). A New Lightweight Stream Cipher Based on Chaos. Symmetry.

[23] Nepomuceno E.G., Nardo L.G., Arias-Garcia J., Butusov D.N., Tutueva A. (2019). Image encryption based on the pseudo-orbits from 1D chaotic map. Chaos Interdiscip. J. Nonlinear Sci..

[24] Zhou Y., Hua Z., Pun C., Chen C.L.P. (2015). Cascade Chaotic System With Applications. IEEE Trans. Cybern..

[25] Tirdad K., Sadeghian A. Hopfield neural networks as pseudo random number generators. Proceedings of the 2010 Annual Meeting of the North American Fuzzy Information Processing Society.

[26] Li Q., Yang X. (2005). Complex dynamics in a simple Hopfield-type neural network. International Symposium on Neural Networks.

[27] Huang Y., Yang X.S. (2006). Chaos and bifurcation in a new class of simple Hopfield neural network. International Symposium on Neural Networks.

[28] Tsaneva-Atanasova K., Osinga H.M., Rieß T., Sherman A. (2010). Full system bifurcation analysis of endocrine bursting models. J. Theor. Biol..

[29] Hindmarsh J.L., Rose R. (1984). A model of neuronal bursting using three coupled first order differential equations. Proc. R. Soc. Lond. Ser. B. Biol. Sci..

[30] Silva-Juarez A., Rodriguez-Gomez G., Fraga L.G.d.l., Guillen-Fernandez O., Tlelo-Cuautle E. (2019). Optimizing the Kaplan–Yorke Dimension of Chaotic Oscillators Applying DE and PSO. Technologies.

[31] Tlelo-Cuautle E., de la Fraga L., Rangel-Magdaleno J. (2016). Engineering Applications of FPGAs.

[32] Rukhin A., Soto J., Nechvatal J., Smid M., Barker E. (2001). A Statistical Test Suite for Random and Pseudorandom Number Generators for Cryptographic Applications.

[33] Bassham L., Rukhin A., Soto J., Nechvatal J., Smid M., Barker E., Leigh S., Levenson M., Vangel M., Banks D. (2010). A Statistical Test Suite for Random and Pseudorandom Number Generators for Cryptographic Applications.

[34] Lu L., Bao C., Ge M., Xu Y., Yang L., Zhan X., Jia Y. (2019). Phase noise-induced coherence resonance in three dimension memristive Hindmarsh-Rose neuron model. Eur. Phys. J. Spec. Top..

[35] Zhang W., Zhu Z., Yu H. (2019). A symmetric image encryption algorithm based on a coupled logistic–bernoulli map and cellular automata diffusion strategy. Entropy.

[36] Lu Q., Zhu C., Deng X. An Efficient Image Encryption Scheme Based on the LSS Chaotic Map and Single S-Box. https://ieeexplore.ieee.org/abstract/document/8977567.

[37] Moafimadani S.S., Chen Y., Tang C. (2019). A New Algorithm for Medical Color Images Encryption Using Chaotic Systems. Entropy.

[38] Wu Y. (2011). NPCR and UACI Randomness Tests for Image Encryption. Cyber J. J. Sel. Areas Telecommun..

